# Supplementary material for: Hematological malignancy burden in mainland China and Taiwan from 1990 to 2021 and decadal projections: Insights from the global burden of disease study 2021
Source: PLoS One. 2025 Jul 21;20(7):e0328526. doi: 10.1371/journal.pone.0328526 (PMC12279097; doi:10.1371/journal.pone.0328526)
Supplement: S1 Fig — (DOCX) [file pone.0328526.s001.docx]

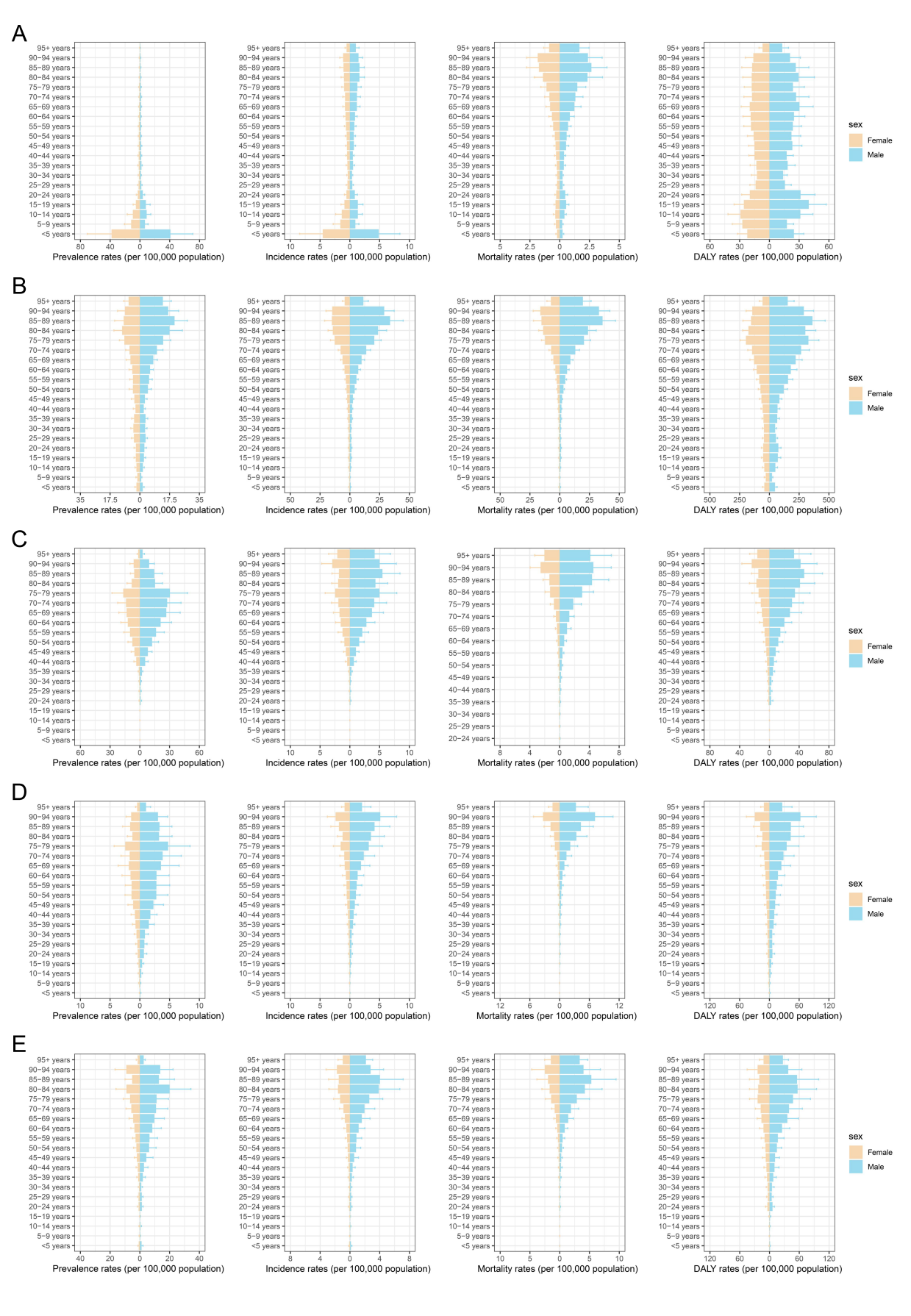


**S1 Fig. Age- and sex-specific distribution of prevalence, incidence, mortality, and DALY rates for leukemia in Taiwan province.**

(A) Distributions of age-standardized prevalence rates (ASPR), incidence rates (ASIR), mortality rates (ASMR), and DALY rates (ASDR) for acute lymphoid leukemia (ALL). (B) Distributions of ASPR, ASIR, ASMR, ASDR for acute myeloid leukemia (AML). (C) Distributions of ASPR, ASIR, ASMR, ASDR for chronic lymphoid leukemia (CLL). (D) Distributions of ASPR, ASIR, ASMR, ASDR for chronic myeloid leukemia (CML). (E) Distributions of ASPR, ASIR, ASMR, ASDR for other leukemia.
