# Supplementary material for: Hematological malignancy burden in mainland China and Taiwan from 1990 to 2021 and decadal projections: Insights from the global burden of disease study 2021
Source: PLoS One. 2025 Jul 21;20(7):e0328526. doi: 10.1371/journal.pone.0328526 (PMC12279097; doi:10.1371/journal.pone.0328526)
Supplement: S2 Fig — (DOCX) [file pone.0328526.s002.docx]

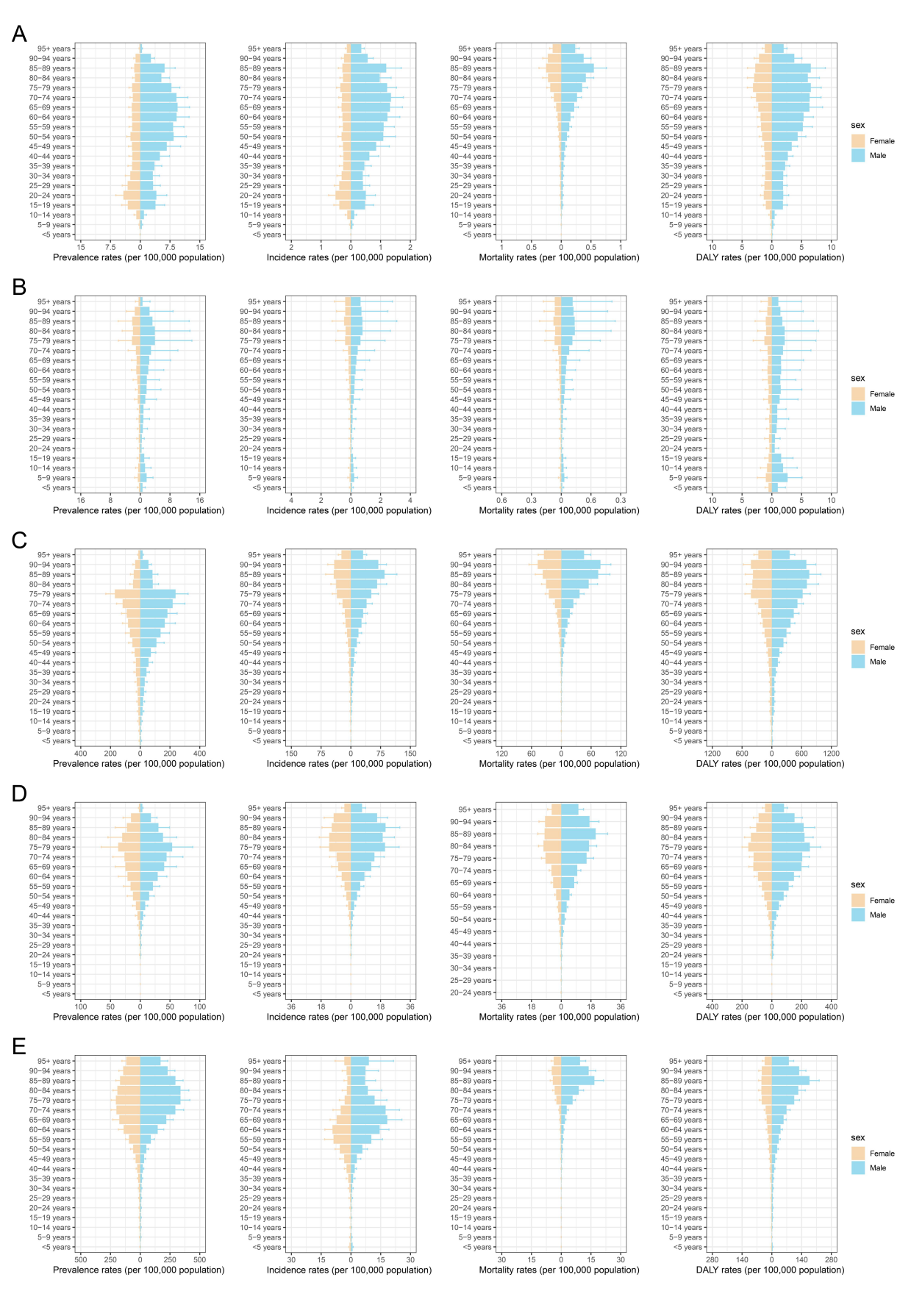


**S2 Fig. Age- and sex-specific distribution of prevalence, incidence, mortality, and DALY rates for lymphoma, multiple myeloma, and other hematological neoplasms in Taiwan province.**

(A) Distributions of age-standardized prevalence rates (ASPR), incidence rates (ASIR), mortality rates (ASMR), and DALY rates (ASDR) for Hodgkin lymphoma (HL). (B) Distributions of ASPR, ASIR, ASMR, ASDR for Burkitt lymphoma (BL). (C) Distributions of ASPR, ASIR, ASMR, ASDR for other non-Hodgkin lymphoma (NHL). (D) Distributions of ASPR, ASIR, ASMR, ASDR for multiple myeloma (MM). (E) Distributions of ASPR, ASIR, ASMR, ASDR for myelodysplastic, myeloproliferative (MD/MP), and other hematopoietic neoplasms.
