# Supplementary material for: Hematological malignancy burden in mainland China and Taiwan from 1990 to 2021 and decadal projections: Insights from the global burden of disease study 2021
Source: PLoS One. 2025 Jul 21;20(7):e0328526. doi: 10.1371/journal.pone.0328526 (PMC12279097; doi:10.1371/journal.pone.0328526)
Supplement: S4 Fig — Temporal trends of disease burden for lymphoma, multiple myeloma, and other hematological neoplasms in mainland China by sex (1990 − 2021). (DOCX) [file pone.0328526.s004.docx]

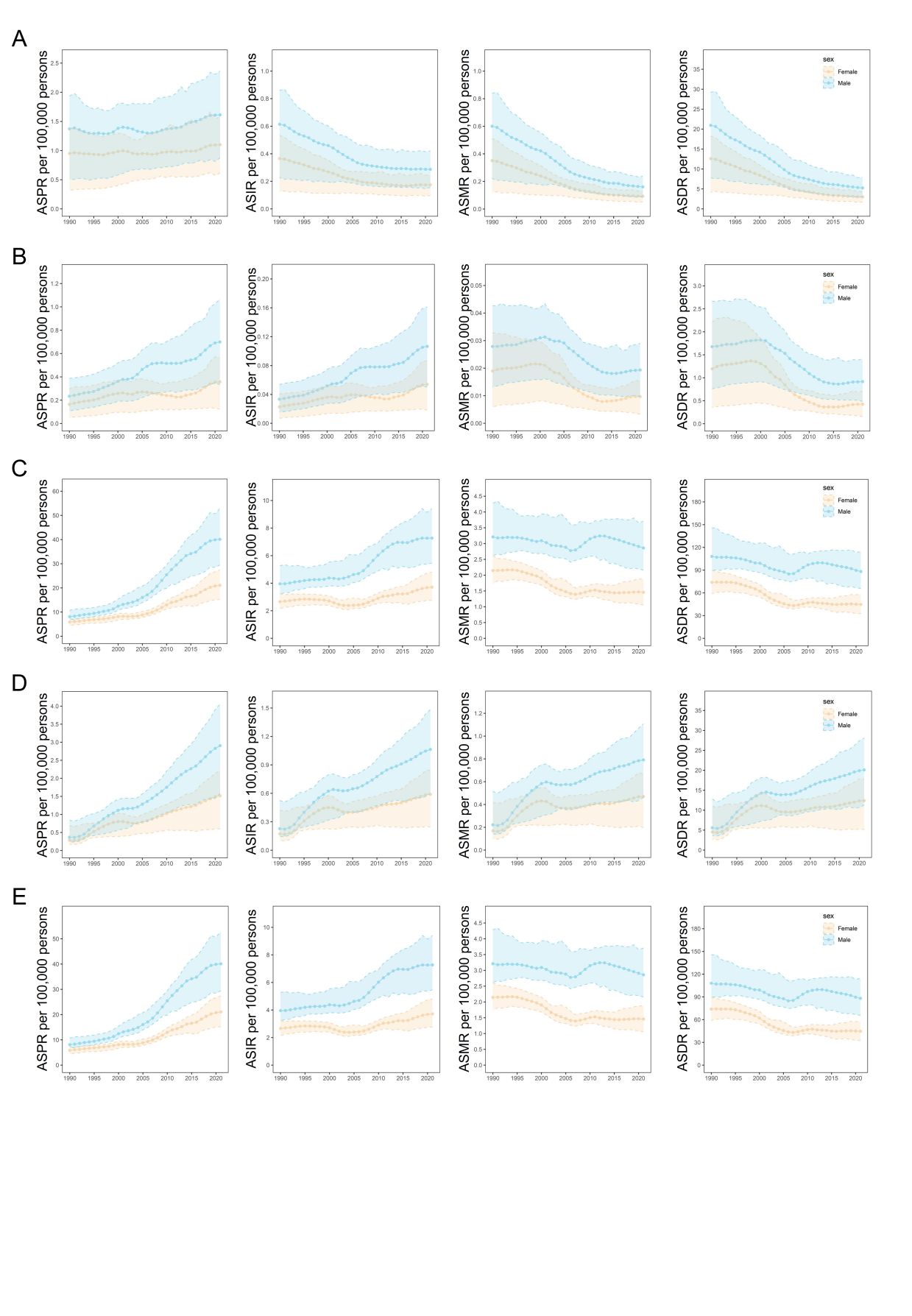


**S4 Fig. Temporal trends of disease burden for lymphoma, multiple myeloma, and other hematological neoplasms in mainland China by sex (1990−2021).**

(A) Temporal trends of age-standardized prevalence rates (ASPR), incidence rates (ASIR), mortality rates (ASMR), and DALY rates (ASDR) for Hodgkin lymphoma (HL). (B) Temporal trends of ASPR, ASIR, ASMR, ASDR for Burkitt lymphoma (BL). (C) Temporal trends of ASPR, ASIR, ASMR, ASDR for other non-Hodgkin lymphoma (NHL) . (D) Temporal trends of ASPR, ASIR, ASMR, ASDR for multiple myeloma (MM). (E) Temporal trends of ASPR, ASIR, ASMR, ASDR for and myelodysplastic, myeloproliferative (MD/MP), and other hematopoietic neoplasms.
