# Supplementary material for: Hematological malignancy burden in mainland China and Taiwan from 1990 to 2021 and decadal projections: Insights from the global burden of disease study 2021
Source: PLoS One. 2025 Jul 21;20(7):e0328526. doi: 10.1371/journal.pone.0328526 (PMC12279097; doi:10.1371/journal.pone.0328526)
Supplement: S5 Fig — Temporal trends of disease burden for leukemia in Taiwan province by sex (1990 − 2021). (DOCX) [file pone.0328526.s005.docx]

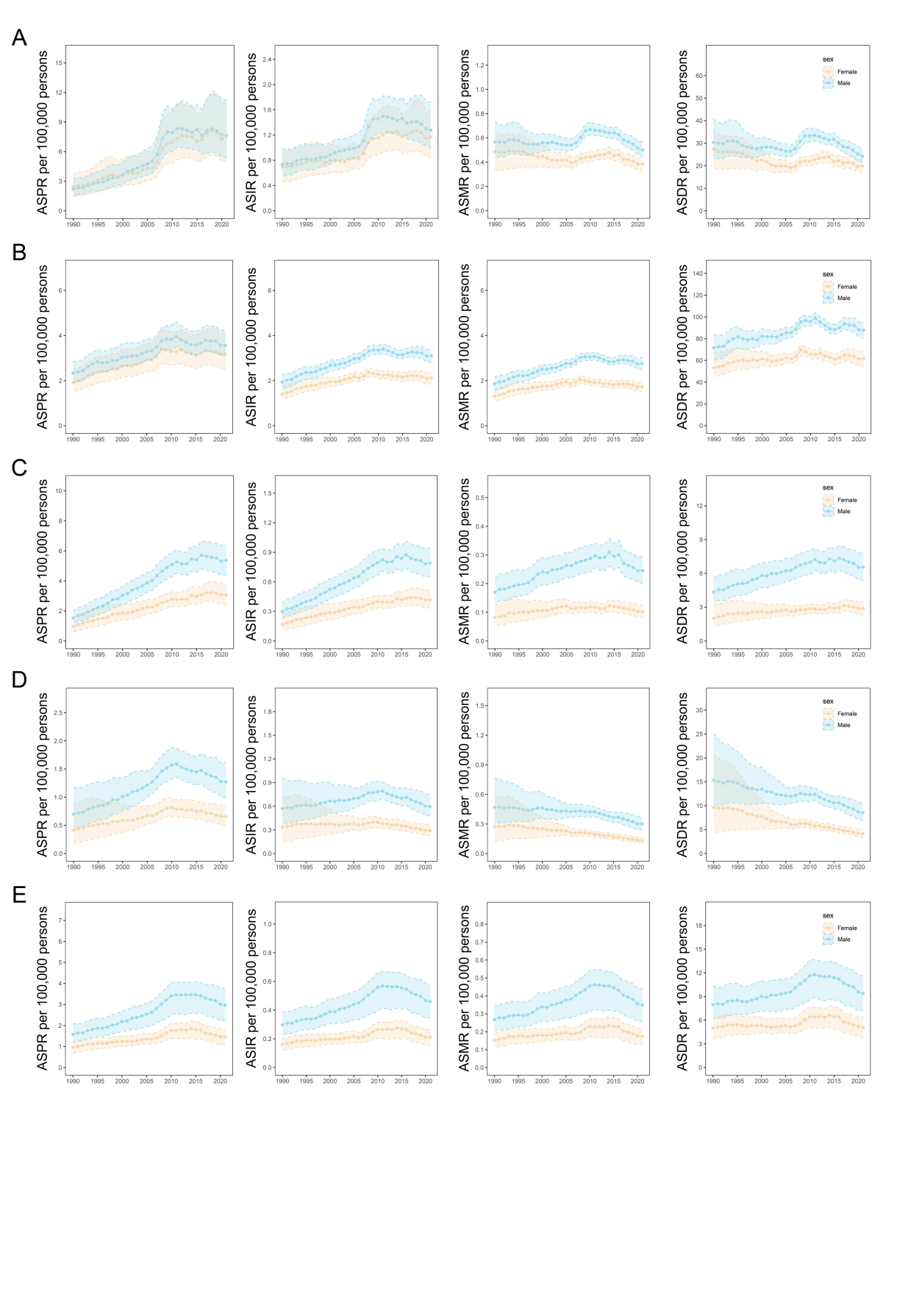


**S5 Fig. Temporal trends of disease burden for leukemia in Taiwan province by sex (1990−2021).**

(A) Temporal trends of age-standardized prevalence rates (ASPR), incidence rates (ASIR), mortality rates (ASMR), and DALY rates (ASDR) for acute lymphoid leukemia (ALL). (B) Temporal trends of ASPR, ASIR, ASMR, ASDR for acute myeloid leukemia (AML). (C) Temporal trends of ASPR, ASIR, ASMR, ASDR for chronic lymphoid leukemia (CLL). (D) Temporal trends of ASPR, ASIR, ASMR, ASDR for chronic myeloid leukemia (CML). (E) Temporal trends of ASPR, ASIR, ASMR, ASDR for other leukemia.
