# Supplementary material for: Hematological malignancy burden in mainland China and Taiwan from 1990 to 2021 and decadal projections: Insights from the global burden of disease study 2021
Source: PLoS One. 2025 Jul 21;20(7):e0328526. doi: 10.1371/journal.pone.0328526 (PMC12279097; doi:10.1371/journal.pone.0328526)
Supplement: S7 Fig — (DOCX) [file pone.0328526.s007.docx]

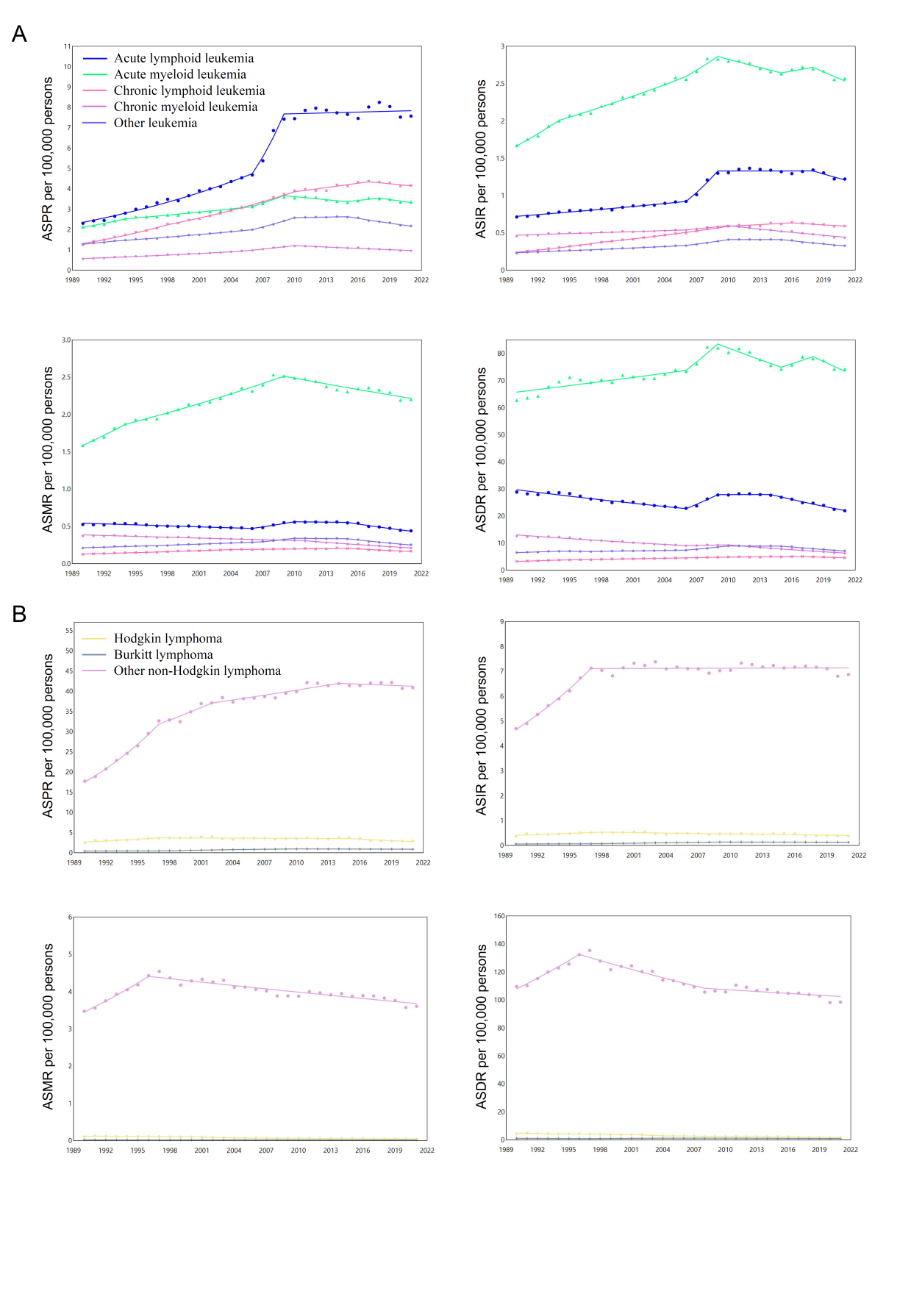


**S7 Fig. Disease burden trends of leukemia and lymphoma analyzed by joinpoint regression analysis in Taiwan province.**

Overall trends of age-standardized prevalence rates (ASPR), incidence rates (ASIR), mortality rates (ASMR), and DALYs rates (ASDR) for leukemia (A) and lymphoma (B) from 1990 to 2021 as indicated.
