# Supplementary material for: Hematological malignancy burden in mainland China and Taiwan from 1990 to 2021 and decadal projections: Insights from the global burden of disease study 2021
Source: PLoS One. 2025 Jul 21;20(7):e0328526. doi: 10.1371/journal.pone.0328526 (PMC12279097; doi:10.1371/journal.pone.0328526)
Supplement: S9 Fig — (DOCX) [file pone.0328526.s009.docx]

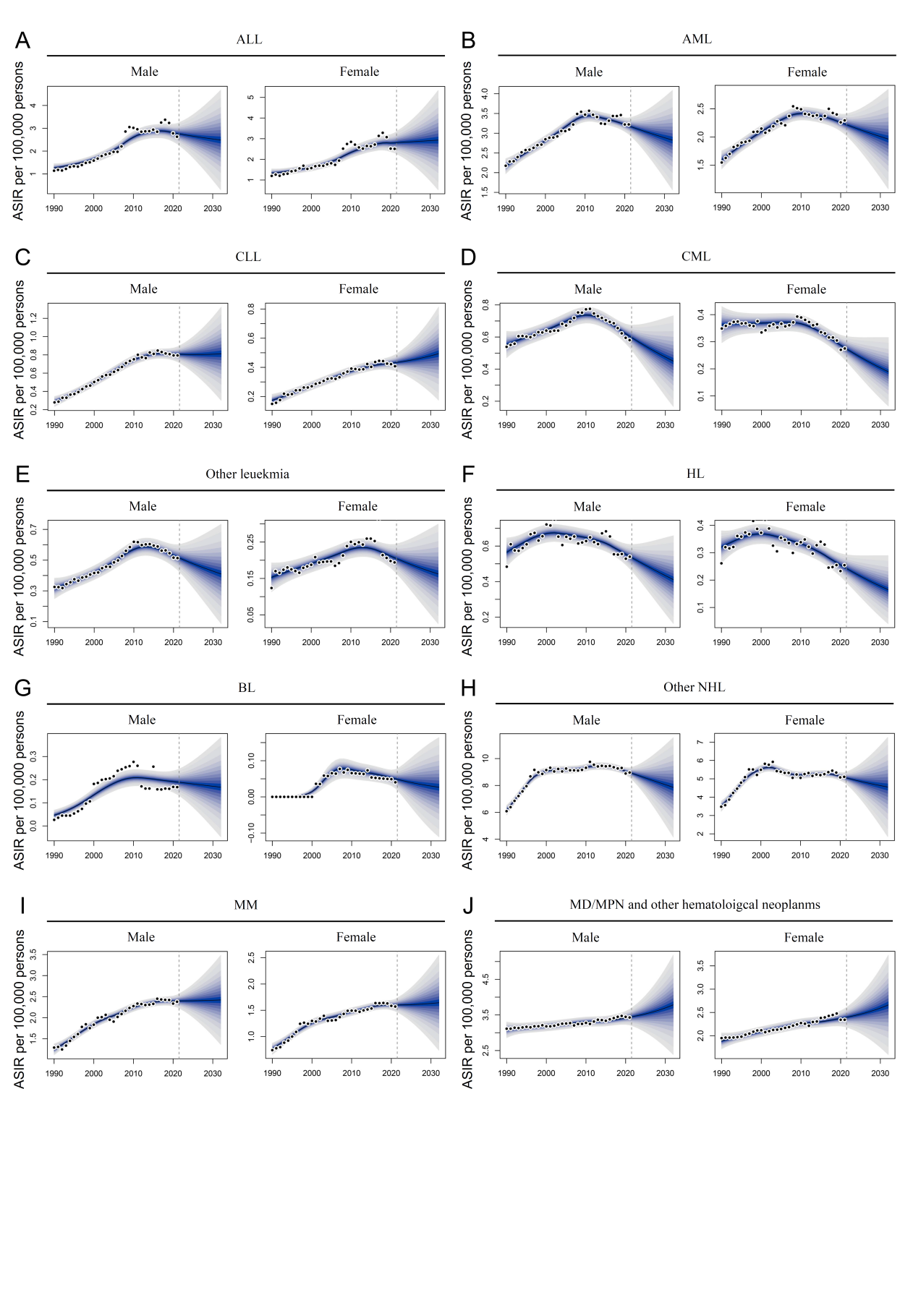


**S9 Fig. Projections of incidence for hematological malignancies in Taiwan province.**

Overall trends of age-standardized incidence rates (ASIR) for acute lymphoid leukemia (ALL) (A), acute myeloid leukemia (AML) (B), chronic lymphoid leukemia (CLL) (C), chronic myeloid leukemia (D), other leukemia (E), Hodgkin lymphoma (HL) (F), Burkitt lymphoma (BL) (G), other non-Hodgkin lymphoma (NHL) (H), multiple myeloma (MM) (I), and myelodysplastic, myeloproliferative (MD/MP), and other hematopoietic neoplasms (J) from 1990 to 2032 as indicated.
