# Supplementary material for: Hematological malignancy burden in mainland China and Taiwan from 1990 to 2021 and decadal projections: Insights from the global burden of disease study 2021
Source: PLoS One. 2025 Jul 21;20(7):e0328526. doi: 10.1371/journal.pone.0328526 (PMC12279097; doi:10.1371/journal.pone.0328526)
Supplement: S1 Table — Temporal joinpoint analysis of ASPR for hematological malignancies in mainland China (1990 − 2021). (DOCX) [file pone.0328526.s011.docx]

**S1 Table Temporal joinpoint analysis of ASPR for hematological malignancies in mainland China (1990−2021).**

| Diseases | Start | End | Values | \|Lower | Upper | P | Measures |
| --- | --- | --- | --- | --- | --- | --- | --- |
| ALL | 1990 | 1997 | 2.89 | 2.49 | 3.30 | <0.0001 | APC |
| ALL | 1997 | 2003 | 1.76 | 1.16 | 2.36 | <0.0001 | APC |
| ALL | 2003 | 2008 | 10.73 | 9.88 | 11.59 | <0.0001 | APC |
| ALL | 2008 | 2014 | 5.97 | 5.31 | 6.62 | <0.0001 | APC |
| ALL | 2014 | 2019 | 3.10 | 1.93 | 4.28 | <0.0001 | APC |
| ALL | 2019 | 2021 | −6.58 | −10.59 | −2.39 | 0.0048 | APC |
| AML | 1990 | 2000 | 0.79 | 0.58 | 1.01 | <0.0001 | APC |
| AML | 2000 | 2004 | −1.26 | −2.31 | −0.20 | 0.0233 | APC |
| AML | 2004 | 2007 | −4.24 | −5.98 | −2.47 | 0.0001 | APC |
| AML | 2007 | 2010 | −2.37 | −4.07 | −0.63 | 0.0109 | APC |
| AML | 2010 | 2014 | −3.68 | −4.55 | −2.8 | <0.0001 | APC |
| AML | 2014 | 2021 | −0.09 | −0.35 | 0.16 | 0.4480 | APC |
| CLL | 1990 | 1996 | 2.94 | 2.55 | 3.33 | <0.0001 | APC |
| CLL | 1996 | 2002 | 4.90 | 4.40 | 5.40 | <0.0001 | APC |
| CLL | 2002 | 2005 | 8.09 | 5.74 | 10.49 | <0.0001 | APC |
| CLL | 2005 | 2012 | 5.42 | 5.02 | 5.81 | <0.0001 | APC |
| CLL | 2012 | 2021 | 2.73 | 2.52 | 2.94 | <0.0001 | APC |
| CML | 1990 | 2002 | 0.03 | −0.20 | 0.27 | 0.7863 | APC |
| CML | 2002 | 2010 | 1.07 | 0.61 | 1.53 | 0.0001 | APC |
| CML | 2010 | 2016 | −0.30 | −1.14 | 0.55 | 0.4741 | APC |
| CML | 2016 | 2021 | 3.88 | 2.92 | 4.86 | <0.0001 | APC |
| Other leukemia | 1990 | 1997 | −0.29 | −0.56 | −0.02 | 0.0367 | APC |
| Other leukemia | 1997 | 2001 | 1.16 | 0.13 | 2.19 | 0.0285 | APC |
| Other leukemia | 2001 | 2011 | 0.25 | 0.06 | 0.44 | 0.0136 | APC |
| Other leukemia | 2011 | 2021 | −0.40 | −0.57 | −0.22 | 0.0001 | APC |
| HL | 1990 | 1997 | −0.88 | −1.26 | −0.50 | 0.0002 | APC |
| HL | 1997 | 2001 | 2.21 | 0.91 | 3.52 | 0.0024 | APC |
| HL | 2001 | 2006 | −1.48 | −2.13 | −0.82 | 0.0002 | APC |
| HL | 2006 | 2016 | 1.14 | 0.93 | 1.34 | <0.0001 | APC |
| HL | 2016 | 2019 | 2.71 | 0.42 | 5.05 | 0.0232 | APC |
| HL | 2019 | 2021 | 0.27 | −1.94 | 2.54 | 0.7979 | APC |
| BL | 1990 | 2000 | 4.65 | 4.25 | 5.06 | <0.0001 | APC |
| BL | 2000 | 2003 | 1.73 | −2.75 | 6.41 | 0.4337 | APC |
| BL | 2003 | 2006 | 6.15 | 1.46 | 11.04 | 0.0124 | APC |
| BL | 2006 | 2014 | −0.30 | −0.84 | 0.23 | 0.2450 | APC |
| BL | 2014 | 2021 | 5.39 | 4.74 | 6.04 | <0.0001 | APC |
| Other NHL | 1990 | 1998 | 3.37 | 3.05 | 3.69 | <0.0001 | APC |
| Other NHL | 1998 | 2001 | 5.69 | 3.06 | 8.39 | 0.0003 | APC |
| Other NHL | 2001 | 2005 | 3.11 | 1.49 | 4.77 | 0.0009 | APC |
| Other NHL | 2005 | 2011 | 9.48 | 8.81 | 10.15 | <0.0001 | APC |
| Other NHL | 2011 | 2014 | 5.78 | 2.60 | 9.06 | 0.0013 | APC |
| Other NHL | 2014 | 2021 | 3.44 | 2.92 | 3.95 | <0.0001 | APC |
| MM | 1990 | 1992 | 2.16 | −3.26 | 7.89 | 0.4165 | APC |
| MM | 1992 | 1995 | 22.12 | 17.84 | 26.56 | <0.0001 | APC |
| MM | 1995 | 1999 | 11.43 | 10.08 | 12.79 | <0.0001 | APC |
| MM | 1999 | 2004 | 0.66 | −0.13 | 1.46 | 0.0969 | APC |
| MM | 2004 | 2012 | 6.01 | 5.71 | 6.31 | <0.0001 | APC |
| MM | 2012 | 2021 | 4.05 | 3.80 | 4.30 | <0.0001 | APC |
| MD/MP & other HM | 1990 | 2005 | 0.25 | 0.24 | 0.27 | <0.0001 | APC |
| MD/MP & other HM | 2005 | 2010 | 3.18 | 3.08 | 3.28 | <0.0001 | APC |
| MD/MP & other HM | 2010 | 2019 | −0.37 | −0.40 | −0.34 | <0.0001 | APC |
| MD/MP & other HM | 2019 | 2021 | −2.37 | −2.65 | −2.09 | <0.0001 | APC |
| ALL | 1990 | 2021 | 3.87 | 3.49 | 4.26 | <0.0001 | AAPC |
| AML | 1990 | 2021 | −1.06 | −1.35 | −0.77 | <0.0001 | AAPC |
| CLL | 1990 | 2021 | 4.30 | 4.04 | 4.56 | <0.0001 | AAPC |
| CML | 1990 | 2021 | 0.85 | 0.59 | 1.10 | <0.0001 | AAPC |
| Other leukemia | 1990 | 2021 | 0.04 | −0.12 | 0.19 | 0.6614 | AAPC |
| HL | 1990 | 2021 | 0.48 | 0.17 | 0.80 | 0.0028 | AAPC |
| BL | 1990 | 2021 | 3.37 | 2.74 | 4.01 | <0.0001 | AAPC |
| Other NHL | 1990 | 2021 | 4.96 | 4.51 | 5.42 | <0.0001 | AAPC |
| MM | 1990 | 2021 | 6.43 | 5.90 | 6.96 | <0.0001 | AAPC |
| MD/MP & other HM | 1990 | 2021 | 0.36 | 0.34 | 0.39 | <0.0001 | AAPC |

ASPR: age-standardized prevalence rates; ALL: acute lymphoid leukemia; AML: acute myeloid leukemia, CLL: chronic lymphoid leukemia; CML: chronic myeloid leukemia; HL: Hodgkin lymphoma; BL: Burkitt lymphoma; NHL: non-Hodgkin lymphoma; MM: multiple myeloma; MD/MP & other HN: myelodysplastic, myeloproliferative, and other hematopoietic neoplasms; ASR: age-standardized rates; APC: annual percent change; AAPC: average annual percent change.
