# Supplementary material for: Hematological malignancy burden in mainland China and Taiwan from 1990 to 2021 and decadal projections: Insights from the global burden of disease study 2021
Source: PLoS One. 2025 Jul 21;20(7):e0328526. doi: 10.1371/journal.pone.0328526 (PMC12279097; doi:10.1371/journal.pone.0328526)
Supplement: S2 Table — Temporal joinpoint analysis of ASIR for hematological malignancies in mainland China (1990 − 2021). (DOCX) [file pone.0328526.s012.docx]

**S2 Table Temporal joinpoint analysis of ASIR for hematological malignancies in mainland China (1990−2021).**

| Diseases | Start | End | Values | \|Lower | Upper | P | Measures |
| --- | --- | --- | --- | --- | --- | --- | --- |
| ALL | 1990 | 1997 | −0.65 | −1.06 | −0.24 | 0.0040 | APC |
| ALL | 1997 | 2004 | −1.81 | −2.27 | −1.36 | <0.0001 | APC |
| ALL | 2004 | 2010 | 3.27 | 2.64 | 3.91 | <0.0001 | APC |
| ALL | 2010 | 2019 | 1.72 | 1.35 | 2.08 | <0.0001 | APC |
| ALL | 2019 | 2021 | −6.18 | −10.12 | −2.06 | 0.0059 | APC |
| AML | 1990 | 2000 | 0.01 | −0.15 | 0.17 | 0.8538 | APC |
| AML | 2000 | 2004 | −1.16 | −2.00 | −0.31 | 0.0097 | APC |
| AML | 2004 | 2014 | −3.07 | −3.21 | −2.94 | <0.0001 | APC |
| AML | 2014 | 2021 | 0.23 | 0.02 | 0.44 | 0.0362 | APC |
| CLL | 1990 | 1997 | 0.94 | 0.65 | 1.24 | <0.0001 | APC |
| CLL | 1997 | 2013 | 3.04 | 2.94 | 3.13 | <0.0001 | APC |
| CLL | 2013 | 2021 | 1.83 | 1.59 | 2.08 | <0.0001 | APC |
| CML | 1990 | 2004 | −1.50 | −1.65 | −1.34 | <0.0001 | APC |
| CML | 2004 | 2007 | −5.82 | −8.40 | −3.17 | 0.0003 | APC |
| CML | 2007 | 2010 | −1.77 | −4.50 | 1.03 | 0.1993 | APC |
| CML | 2010 | 2015 | −3.91 | −4.88 | −2.93 | <0.0001 | APC |
| CML | 2015 | 2021 | 1.11 | 0.51 | 1.72 | 0.0010 | APC |
| Other leukemia | 1990 | 1997 | −1.06 | −1.23 | −0.89 | <0.0001 | APC |
| Other leukemia | 1997 | 2004 | −0.29 | −0.51 | −0.07 | 0.0119 | APC |
| Other leukemia | 2004 | 2007 | −1.78 | −3.13 | −0.4 | 0.0148 | APC |
| Other leukemia | 2007 | 2010 | 0.37 | −1.02 | 1.78 | 0.5815 | APC |
| Other leukemia | 2010 | 2015 | −1.50 | −1.92 | −1.08 | <0.0001 | APC |
| Other leukemia | 2015 | 2021 | −0.89 | −1.12 | −0.66 | <0.0001 | APC |
| HL | 1990 | 2000 | −3.03 | −3.16 | −2.9 | <0.0001 | APC |
| HL | 2000 | 2007 | −4.76 | −4.98 | −4.53 | <0.0001 | APC |
| HL | 2007 | 2015 | −1.45 | −1.63 | −1.26 | <0.0001 | APC |
| HL | 2015 | 2021 | 0.24 | 0.01 | 0.48 | 0.0426 | APC |
| BL | 1990 | 2007 | 4.50 | 4.26 | 4.73 | <0.0001 | APC |
| BL | 2007 | 2013 | −0.78 | −1.91 | 0.36 | 0.1719 | APC |
| BL | 2013 | 2021 | 4.94 | 4.24 | 5.65 | <0.0001 | APC |
| Other NHL | 1990 | 1995 | 1.39 | 0.96 | 1.83 | <0.0001 | APC |
| Other NHL | 1995 | 2000 | 0.03 | −0.45 | 0.51 | 0.8982 | APC |
| Other NHL | 2000 | 2003 | −1.83 | −3.56 | −0.07 | 0.0426 | APC |
| Other NHL | 2003 | 2006 | 1.83 | −0.35 | 4.07 | 0.0943 | APC |
| Other NHL | 2006 | 2012 | 5.62 | 5.22 | 6.03 | <0.0001 | APC |
| Other NHL | 2012 | 2021 | 1.29 | 1.09 | 1.50 | <0.0001 | APC |
| MM | 1990 | 1992 | 1.48 | −4.30 | 7.61 | 0.6011 | APC |
| MM | 1992 | 1995 | 21.14 | 16.55 | 25.92 | <0.0001 | APC |
| MM | 1995 | 1999 | 10.35 | 9.00 | 11.73 | <0.0001 | APC |
| MM | 1999 | 2005 | −0.77 | −1.32 | −0.22 | 0.0090 | APC |
| MM | 2005 | 2011 | 3.76 | 3.26 | 4.26 | <0.0001 | APC |
| MM | 2011 | 2021 | 2.64 | 2.44 | 2.85 | <0.0001 | APC |
| MD/MP & other HM | 1990 | 2003 | 0.27 | 0.26 | 0.28 | <0.0001 | APC |
| MD/MP & other HM | 2003 | 2006 | 1.15 | 1.00 | 1.30 | <0.0001 | APC |
| MD/MP & other HM | 2006 | 2009 | 5.97 | 5.83 | 6.12 | <0.0001 | APC |
| MD/MP & other HM | 2009 | 2012 | 0.32 | 0.19 | 0.45 | 0.0001 | APC |
| MD/MP & other HM | 2012 | 2019 | −0.47 | −0.49 | −0.45 | <0.0001 | APC |
| MD/MP & other HM | 2019 | 2021 | −1.64 | −1.77 | −1.52 | <0.0001 | APC |
| ALL | 1990 | 2021 | 0.15 | −0.18 | 0.47 | 0.3817 | AAPC |
| AML | 1990 | 2021 | −1.10 | −1.22 | −0.97 | <0.0001 | AAPC |
| CLL | 1990 | 2021 | 2.25 | 2.15 | 2.35 | <0.0001 | AAPC |
| CML | 1990 | 2021 | −1.84 | −2.24 | −1.44 | <0.0001 | AAPC |
| Other leukemia | 1990 | 2021 | −0.86 | −1.05 | −0.66 | <0.0001 | AAPC |
| HL | 1990 | 2021 | −2.39 | −2.48 | −2.31 | <0.0001 | AAPC |
| BL | 1990 | 2021 | 3.57 | 3.27 | 3.87 | <0.0001 | AAPC |
| Other NHL | 1990 | 2021 | 1.67 | 1.39 | 1.96 | <0.0001 | AAPC |
| MM | 1990 | 2021 | 4.74 | 4.18 | 5.29 | <0.0001 | AAPC |
| MD/MP & other HM | 1990 | 2021 | 0.60 | 0.58 | 0.63 | <0.0001 | AAPC |

ASIR: age-standardized incidence rates; ALL: acute lymphoid leukemia; AML: acute myeloid leukemia, CLL: chronic lymphoid leukemia; CML: chronic myeloid leukemia; HL: Hodgkin lymphoma; BL: Burkitt lymphoma; NHL: non-Hodgkin lymphoma; MM: multiple myeloma; MD/MP & other HN: myelodysplastic, myeloproliferative, and other hematopoietic neoplasms; ASR: age-standardized rates; APC: annual percent change; AAPC: average annual percent change.
