# Supplementary material for: Hematological malignancy burden in mainland China and Taiwan from 1990 to 2021 and decadal projections: Insights from the global burden of disease study 2021
Source: PLoS One. 2025 Jul 21;20(7):e0328526. doi: 10.1371/journal.pone.0328526 (PMC12279097; doi:10.1371/journal.pone.0328526)
Supplement: S3 Table — Temporal joinpoint analysis of ASMR for hematological malignancies in mainland China (1990 − 2021). (DOCX) [file pone.0328526.s013.docx]

**S3 Table Temporal joinpoint analysis of ASMR for hematological malignancies in mainland China (1990−2021).**

| Diseases | Start | End | Values | \|Lower | Upper | P | Measures |
| --- | --- | --- | --- | --- | --- | --- | --- |
| ALL | 1990 | 1993 | −1.33 | −2.34 | −0.31 | 0.0138 | APC |
| ALL | 1993 | 2000 | −2.37 | −2.69 | −2.05 | <0.0001 | APC |
| ALL | 2000 | 2006 | −4.26 | −4.68 | −3.84 | <0.0001 | APC |
| ALL | 2006 | 2009 | −0.82 | −2.67 | 1.05 | 0.3658 | APC |
| ALL | 2009 | 2021 | −2.55 | −2.68 | −2.43 | <0.0001 | APC |
| AML | 1990 | 2000 | −0.19 | −0.29 | −0.09 | 0.0010 | APC |
| AML | 2000 | 2004 | −1.37 | −1.88 | −0.86 | <0.0001 | APC |
| AML | 2004 | 2007 | −4.49 | −5.39 | −3.59 | <0.0001 | APC |
| AML | 2007 | 2010 | −3.23 | −4.16 | −2.3 | <0.0001 | APC |
| AML | 2010 | 2014 | −3.92 | −4.37 | −3.47 | <0.0001 | APC |
| AML | 2014 | 2021 | −0.37 | −0.50 | −0.23 | <0.0001 | APC |
| CLL | 1990 | 1997 | −0.55 | −0.73 | −0.37 | <0.0001 | APC |
| CLL | 1997 | 2000 | 0.84 | −0.44 | 2.15 | 0.1849 | APC |
| CLL | 2000 | 2004 | −0.19 | −0.84 | 0.46 | 0.5486 | APC |
| CLL | 2004 | 2007 | −3.43 | −4.75 | −2.09 | <0.0001 | APC |
| CLL | 2007 | 2021 | −0.70 | −0.76 | −0.64 | <0.0001 | APC |
| CML | 1990 | 2001 | −2.29 | −2.41 | −2.17 | <0.0001 | APC |
| CML | 2001 | 2004 | −4.19 | −5.75 | −2.62 | 0.0001 | APC |
| CML | 2004 | 2007 | −9.79 | −11.13 | −8.43 | <0.0001 | APC |
| CML | 2007 | 2010 | −4.66 | −6.13 | −3.15 | <0.0001 | APC |
| CML | 2010 | 2015 | −6.79 | −7.33 | −6.25 | <0.0001 | APC |
| CML | 2015 | 2021 | −1.51 | −1.85 | −1.16 | <0.0001 | APC |
| Other leukemia | 1990 | 1997 | −1.88 | −2.02 | −1.75 | <0.0001 | APC |
| Other leukemia | 1997 | 2004 | −0.67 | −0.84 | −0.51 | <0.0001 | APC |
| Other leukemia | 2004 | 2007 | −3.38 | −4.37 | −2.39 | <0.0001 | APC |
| Other leukemia | 2007 | 2010 | −0.43 | −1.43 | 0.57 | 0.3710 | APC |
| Other leukemia | 2010 | 2014 | −2.19 | −2.65 | −1.73 | <0.0001 | APC |
| Other leukemia | 2014 | 2021 | −1.32 | −1.45 | −1.18 | <0.0001 | APC |
| HL | 1990 | 2001 | −3.77 | −3.92 | −3.62 | <0.0001 | APC |
| HL | 2001 | 2007 | −7.45 | −7.82 | −7.08 | <0.0001 | APC |
| HL | 2007 | 2014 | −4.29 | −4.59 | −4.00 | <0.0001 | APC |
| HL | 2014 | 2021 | −2.03 | −2.28 | −1.79 | <0.0001 | APC |
| BL | 1990 | 2001 | 1.12 | 0.95 | 1.28 | <0.0001 | APC |
| BL | 2001 | 2005 | −3.64 | −4.69 | −2.57 | <0.0001 | APC |
| BL | 2005 | 2010 | −7.04 | −7.62 | −6.47 | <0.0001 | APC |
| BL | 2010 | 2013 | −5.76 | −7.51 | −3.98 | <0.0001 | APC |
| BL | 2013 | 2016 | 0.16 | −1.97 | 2.34 | 0.8749 | APC |
| BL | 2016 | 2021 | 2.17 | 1.65 | 2.69 | <0.0001 | APC |
| Other NHL | 1990 | 1995 | 0.06 | −0.12 | 0.24 | 0.5118 | APC |
| Other NHL | 1995 | 2000 | −1.51 | −1.71 | −1.32 | <0.0001 | APC |
| Other NHL | 2000 | 2003 | −3.50 | −4.21 | −2.79 | <0.0001 | APC |
| Other NHL | 2003 | 2007 | −1.89 | −2.32 | −1.47 | <0.0001 | APC |
| Other NHL | 2007 | 2011 | 3.49 | 3.14 | 3.85 | <0.0001 | APC |
| Other NHL | 2011 | 2021 | −0.98 | −1.05 | −0.91 | <0.0001 | APC |
| MM | 1990 | 1992 | 1.39 | −8.39 | 12.22 | 0.7755 | APC |
| MM | 1992 | 1995 | 20.44 | 12.56 | 28.88 | <0.0001 | APC |
| MM | 1995 | 1998 | 10.81 | 6.43 | 15.38 | 0.0001 | APC |
| MM | 1998 | 2001 | 2.88 | −1.96 | 7.95 | 0.2288 | APC |
| MM | 2001 | 2004 | −3.80 | −7.74 | 0.31 | 0.0671 | APC |
| MM | 2004 | 2021 | 1.95 | 1.80 | 2.10 | <0.0001 | APC |
| MD/MP & other HM | 1990 | 1998 | 1.41 | 1.27 | 1.55 | <0.0001 | APC |
| MD/MP & other HM | 1998 | 2010 | 2.55 | 2.48 | 2.63 | <0.0001 | APC |
| MD/MP & other HM | 2010 | 2015 | −0.10 | −0.42 | 0.21 | 0.5017 | APC |
| MD/MP & other HM | 2015 | 2021 | 0.98 | 0.81 | 1.15 | <0.0001 | APC |
| ALL | 1990 | 2021 | −2.56 | −2.78 | −2.34 | <0.0001 | AAPC |
| AML | 1990 | 2021 | −1.59 | −1.74 | −1.44 | <0.0001 | AAPC |
| CLL | 1990 | 2021 | −0.72 | −0.91 | −0.53 | <0.0001 | AAPC |
| CML | 1990 | 2021 | −4.04 | −4.30 | −3.78 | <0.0001 | AAPC |
| Other leukemia | 1990 | 2021 | −1.53 | −1.68 | −1.38 | <0.0001 | AAPC |
| HL | 1990 | 2021 | −4.23 | −4.34 | −4.11 | <0.0001 | AAPC |
| BL | 1990 | 2021 | −1.46 | −1.77 | −1.15 | <0.0001 | AAPC |
| Other NHL | 1990 | 2021 | −0.70 | −0.80 | −0.60 | <0.0001 | AAPC |
| MM | 1990 | 2021 | 3.91 | 2.79 | 5.05 | <0.0001 | AAPC |
| MD/MP & other HM | 1990 | 2021 | 1.52 | 1.45 | 1.59 | <0.0001 | AAPC |

ASMR: age-standardized mortality rates; ALL: acute lymphoid leukemia; AML: acute myeloid leukemia, CLL: chronic lymphoid leukemia; CML: chronic myeloid leukemia; HL: Hodgkin lymphoma; BL: Burkitt lymphoma; NHL: non-Hodgkin lymphoma; MM: multiple myeloma; MD/MP & other HN: myelodysplastic, myeloproliferative, and other hematopoietic neoplasms; ASR: age-standardized rates; APC: annual percent change; AAPC: average annual percent change.
