# Supplementary material for: Hematological malignancy burden in mainland China and Taiwan from 1990 to 2021 and decadal projections: Insights from the global burden of disease study 2021
Source: PLoS One. 2025 Jul 21;20(7):e0328526. doi: 10.1371/journal.pone.0328526 (PMC12279097; doi:10.1371/journal.pone.0328526)
Supplement: S4 Table — Temporal joinpoint analysis of ASDR for hematological malignancies in mainland China (1990 − 2021). (DOCX) [file pone.0328526.s014.docx]

**S4 Table Temporal joinpoint analysis of ASDR for hematological malignancies in mainland China (1990−2021).**

| Diseases | Start | End | Values | \|Lower | Upper | P | Measures |
| --- | --- | --- | --- | --- | --- | --- | --- |
| ALL | 1990 | 1999 | −2.20 | −2.48 | −1.92 | <0.0001 | APC |
| ALL | 1999 | 2006 | −5.29 | −5.73 | −4.85 | <0.0001 | APC |
| ALL | 2006 | 2009 | −1.25 | −3.88 | 1.45 | 0.3431 | APC |
| ALL | 2009 | 2021 | −2.98 | −3.16 | −2.80 | <0.0001 | APC |
| AML | 1990 | 2000 | −0.51 | −0.70 | −0.33 | <0.0001 | APC |
| AML | 2000 | 2004 | −2.59 | −3.49 | −1.67 | <0.0001 | APC |
| AML | 2004 | 2007 | −5.84 | −7.40 | −4.26 | <0.0001 | APC |
| AML | 2007 | 2010 | −3.84 | −5.37 | −2.29 | 0.0001 | APC |
| AML | 2010 | 2014 | −4.69 | −5.47 | −3.91 | <0.0001 | APC |
| AML | 2014 | 2021 | −0.92 | −1.14 | −0.69 | <0.0001 | APC |
| CLL | 1990 | 1996 | −0.94 | −1.17 | −0.70 | <0.0001 | APC |
| CLL | 1996 | 2004 | 0.38 | 0.20 | 0.56 | 0.0003 | APC |
| CLL | 2004 | 2007 | −3.52 | −4.86 | −2.17 | <0.0001 | APC |
| CLL | 2007 | 2021 | −0.37 | −0.44 | −0.31 | <0.0001 | APC |
| CML | 1990 | 2000 | −2.54 | −2.72 | −2.36 | <0.0001 | APC |
| CML | 2000 | 2004 | −6.17 | −7.15 | −5.18 | <0.0001 | APC |
| CML | 2004 | 2007 | −10.68 | −12.32 | −9.01 | <0.0001 | APC |
| CML | 2007 | 2010 | −5.02 | −6.90 | −3.11 | 0.0001 | APC |
| CML | 2010 | 2015 | −7.42 | −8.10 | −6.73 | <0.0001 | APC |
| CML | 2015 | 2021 | −1.23 | −1.67 | −0.80 | <0.0001 | APC |
| Other leukemia | 1990 | 1996 | −2.15 | −2.35 | −1.96 | <0.0001 | APC |
| Other leukemia | 1996 | 2004 | −1.27 | −1.42 | −1.12 | <0.0001 | APC |
| Other leukemia | 2004 | 2007 | −3.78 | −4.94 | −2.60 | <0.0001 | APC |
| Other leukemia | 2007 | 2010 | −0.72 | −1.91 | 0.48 | 0.2210 | APC |
| Other leukemia | 2010 | 2013 | −2.86 | −3.98 | −1.72 | 0.0001 | APC |
| Other leukemia | 2013 | 2021 | −1.32 | −1.46 | −1.19 | <0.0001 | APC |
| HL | 1990 | 2001 | −4.06 | −4.19 | −3.92 | <0.0001 | APC |
| HL | 2001 | 2007 | −7.84 | −8.19 | −7.49 | <0.0001 | APC |
| HL | 2007 | 2014 | −4.44 | −4.73 | −4.14 | <0.0001 | APC |
| HL | 2014 | 2021 | −2.25 | −2.49 | −2.00 | <0.0001 | APC |
| BL | 1990 | 2000 | 0.99 | 0.77 | 1.20 | <0.0001 | APC |
| BL | 2000 | 2004 | −5.43 | −6.55 | −4.30 | <0.0001 | APC |
| BL | 2004 | 2012 | −7.65 | −7.91 | −7.39 | <0.0001 | APC |
| BL | 2012 | 2015 | −3.09 | −5.40 | −0.73 | 0.0136 | APC |
| BL | 2015 | 2021 | 1.75 | 1.31 | 2.19 | <0.0001 | APC |
| Other NHL | 1990 | 1995 | −0.21 | −0.44 | 0.01 | 0.0635 | APC |
| Other NHL | 1995 | 2000 | −2.15 | −2.41 | −1.90 | <0.0001 | APC |
| Other NHL | 2000 | 2003 | −4.78 | −5.70 | −3.85 | <0.0001 | APC |
| Other NHL | 2003 | 2007 | −2.10 | −2.66 | −1.54 | <0.0001 | APC |
| Other NHL | 2007 | 2011 | 3.66 | 3.20 | 4.13 | <0.0001 | APC |
| Other NHL | 2011 | 2021 | −1.02 | −1.11 | −0.93 | <0.0001 | APC |
| MM | 1990 | 1992 | 0.88 | −5.09 | 7.23 | 0.7627 | APC |
| MM | 1992 | 1995 | 20.36 | 15.63 | 25.28 | <0.0001 | APC |
| MM | 1995 | 1999 | 9.57 | 8.13 | 11.03 | <0.0001 | APC |
| MM | 1999 | 2006 | −1.42 | −1.85 | −0.99 | <0.0001 | APC |
| MM | 2006 | 2011 | 3.24 | 2.49 | 3.99 | <0.0001 | APC |
| MM | 2011 | 2021 | 1.85 | 1.63 | 2.07 | <0.0001 | APC |
| MD/MP & other HM | 1990 | 1998 | 0.85 | 0.81 | 0.89 | <0.0001 | APC |
| MD/MP & other HM | 1998 | 2005 | 1.27 | 1.21 | 1.33 | <0.0001 | APC |
| MD/MP & other HM | 2005 | 2010 | 2.27 | 2.17 | 2.37 | <0.0001 | APC |
| MD/MP & other HM | 2010 | 2015 | −0.45 | −0.55 | −0.35 | <0.0001 | APC |
| MD/MP & other HM | 2015 | 2019 | 0.59 | 0.42 | 0.75 | <0.0001 | APC |
| MD/MP & other HM | 2019 | 2021 | −0.42 | −0.75 | −0.08 | 0.0186 | APC |
| ALL | 1990 | 2021 | −3.12 | −3.39 | −2.84 | <0.0001 | AAPC |
| AML | 1990 | 2021 | −2.27 | −2.53 | −2.01 | <0.0001 | AAPC |
| CLL | 1990 | 2021 | −0.60 | −0.74 | −0.46 | <0.0001 | AAPC |
| CML | 1990 | 2021 | −4.62 | −4.91 | −4.32 | <0.0001 | AAPC |
| Other leukemia | 1990 | 2021 | −1.80 | −1.99 | −1.61 | <0.0001 | AAPC |
| HL | 1990 | 2021 | −4.48 | −4.60 | −4.37 | <0.0001 | AAPC |
| BL | 1990 | 2021 | −2.40 | −2.68 | −2.12 | <0.0001 | AAPC |
| Other NHL | 1990 | 2021 | −1.00 | −1.13 | −0.86 | <0.0001 | AAPC |
| MM | 1990 | 2021 | 3.89 | 3.31 | 4.46 | <0.0001 | AAPC |
| MD/MP & other HM | 1990 | 2021 | 0.84 | 0.81 | 0.88 | <0.0001 | AAPC |

ASDR: age-standardized DALYs rates; ALL: acute lymphoid leukemia; AML: acute myeloid leukemia, CLL: chronic lymphoid leukemia; CML: chronic myeloid leukemia; HL: Hodgkin lymphoma; BL: Burkitt lymphoma; NHL: non-Hodgkin lymphoma; MM: multiple myeloma; MD/MP & other HN: myelodysplastic, myeloproliferative, and other hematopoietic neoplasms; ASR: age-standardized rates; APC: annual percent change; AAPC: average annual percent change.
