# Supplementary material for: Hematological malignancy burden in mainland China and Taiwan from 1990 to 2021 and decadal projections: Insights from the global burden of disease study 2021
Source: PLoS One. 2025 Jul 21;20(7):e0328526. doi: 10.1371/journal.pone.0328526 (PMC12279097; doi:10.1371/journal.pone.0328526)
Supplement: S5 Table — Temporal joinpoint analysis of ASPR for hematological malignancies in Taiwan (1990 − 2021). (DOCX) [file pone.0328526.s015.docx]

**S5 Table Temporal joinpoint analysis of ASPR for hematological malignancies in Taiwan (1990−2021).**

| Diseases | Start | End | Values | \|Lower | Upper | P | Measures |
| --- | --- | --- | --- | --- | --- | --- | --- |
| ALL | 1990 | 2006 | 4.49 | 4.14 | 4.83 | <0.0001 | APC |
| ALL | 2006 | 2009 | 17.39 | 9.01 | 26.43 | 0.0002 | APC |
| ALL | 2009 | 2021 | 0.17 | −0.30 | 0.65 | 0.4630 | APC |
| AML | 1990 | 1994 | 4.60 | 2.78 | 6.44 | 0.0001 | APC |
| AML | 1994 | 2006 | 1.81 | 1.49 | 2.12 | <0.0001 | APC |
| AML | 2006 | 2009 | 5.50 | 1.28 | 9.89 | 0.0135 | APC |
| AML | 2009 | 2015 | −1.42 | −2.32 | −0.5 | 0.0050 | APC |
| AML | 2015 | 2018 | 1.77 | −2.85 | 6.60 | 0.4341 | APC |
| AML | 2018 | 2021 | −2.16 | −4.40 | 0.14 | 0.0634 | APC |
| CLL | 1990 | 1998 | 6.87 | 6.00 | 7.74 | <0.0001 | APC |
| CLL | 1998 | 2010 | 4.66 | 4.31 | 5.01 | <0.0001 | APC |
| CLL | 2010 | 2017 | 1.72 | 1.00 | 2.46 | 0.0001 | APC |
| CLL | 2017 | 2021 | −1.16 | −2.75 | 0.46 | 0.1517 | APC |
| CML | 1990 | 2005 | 3.34 | 3.04 | 3.63 | <0.0001 | APC |
| CML | 2005 | 2010 | 5.24 | 4.26 | 6.23 | <0.0001 | APC |
| CML | 2010 | 2021 | −1.99 | −2.22 | −1.76 | <0.0001 | APC |
| Other leukemia | 1990 | 1993 | 3.88 | 2.22 | 5.57 | 0.0001 | APC |
| Other leukemia | 1993 | 2006 | 2.56 | 2.39 | 2.74 | <0.0001 | APC |
| Other leukemia | 2006 | 2010 | 6.72 | 5.05 | 8.42 | <0.0001 | APC |
| Other leukemia | 2010 | 2015 | 0.37 | −0.63 | 1.39 | 0.4455 | APC |
| Other leukemia | 2015 | 2021 | −3.19 | −3.76 | −2.63 | <0.0001 | APC |
| HL | 1990 | 1997 | 4.87 | 2.72 | 7.08 | 0.0001 | APC |
| HL | 1997 | 2015 | −0.28 | −0.82 | 0.26 | 0.2929 | APC |
| HL | 2015 | 2021 | −3.93 | −6.98 | −0.78 | 0.0171 | APC |
| BL | 1990 | 1997 | 1.86 | 1.41 | 2.30 | <0.0001 | APC |
| BL | 1997 | 2004 | 7.47 | 6.88 | 8.07 | <0.0001 | APC |
| BL | 2004 | 2010 | 3.16 | 2.28 | 4.05 | <0.0001 | APC |
| BL | 2010 | 2021 | −0.65 | −0.95 | −0.36 | 0.0002 | APC |
| Other NHL | 1990 | 1997 | 8.90 | 8.17 | 9.64 | <0.0001 | APC |
| Other NHL | 1997 | 2002 | 3.07 | 1.52 | 4.64 | 0.0004 | APC |
| Other NHL | 2002 | 2014 | 1.04 | 0.67 | 1.41 | <0.0001 | APC |
| Other NHL | 2014 | 2021 | −0.24 | −1.10 | 0.62 | 0.5648 | APC |
| MM | 1990 | 1992 | 0.83 | −7.01 | 9.34 | 0.8318 | APC |
| MM | 1992 | 1998 | 7.81 | 5.84 | 9.82 | <0.0001 | APC |
| MM | 1998 | 2006 | 1.64 | 0.55 | 2.73 | 0.0051 | APC |
| MM | 2006 | 2009 | 4.96 | −3.79 | 14.50 | 0.2581 | APC |
| MM | 2009 | 2021 | 1.26 | 0.66 | 1.87 | 0.0003 | APC |
| MD/MP & other HM | 1990 | 2000 | 0.36 | 0.35 | 0.38 | <0.0001 | APC |
| MD/MP & other HM | 2000 | 2005 | 0.24 | 0.18 | 0.30 | <0.0001 | APC |
| MD/MP & other HM | 2005 | 2008 | 0.44 | 0.24 | 0.63 | 0.0002 | APC |
| MD/MP & other HM | 2008 | 2019 | 0.25 | 0.23 | 0.26 | <0.0001 | APC |
| MD/MP & other HM | 2019 | 2021 | 0.07 | −0.08 | 0.22 | 0.3671 | APC |
| ALL | 1990 | 2021 | 3.96 | 3.21 | 4.71 | <0.0001 | AAPC |
| AML | 1990 | 2021 | 1.48 | 0.82 | 2.15 | <0.0001 | AAPC |
| CLL | 1990 | 2021 | 3.78 | 3.43 | 4.14 | <0.0001 | AAPC |
| CML | 1990 | 2021 | 1.71 | 1.50 | 1.93 | <0.0001 | AAPC |
| Other leukemia | 1990 | 2021 | 1.72 | 1.40 | 2.03 | <0.0001 | AAPC |
| HL | 1990 | 2021 | 0.14 | −0.66 | 0.94 | 0.7407 | AAPC |
| BL | 1990 | 2021 | 2.44 | 2.20 | 2.69 | <0.0001 | AAPC |
| Other NHL | 1990 | 2021 | 2.80 | 2.43 | 3.16 | <0.0001 | AAPC |
| MM | 1990 | 2021 | 2.92 | 1.86 | 4.00 | <0.0001 | AAPC |
| MD/MP & other HM | 1990 | 2021 | 0.29 | 0.27 | 0.31 | <0.0001 | AAPC |

ASPR: age-standardized prevalence rates; ALL: acute lymphoid leukemia; AML: acute myeloid leukemia, CLL: chronic lymphoid leukemia; CML: chronic myeloid leukemia; HL: Hodgkin lymphoma; BL: Burkitt lymphoma; NHL: non-Hodgkin lymphoma; MM: multiple myeloma; MD/MP & other HN: myelodysplastic, myeloproliferative, and other hematopoietic neoplasms; ASR: age-standardized rates; APC: annual percent change; AAPC: average annual percent change.
