# Supplementary material for: Hematological malignancy burden in mainland China and Taiwan from 1990 to 2021 and decadal projections: Insights from the global burden of disease study 2021
Source: PLoS One. 2025 Jul 21;20(7):e0328526. doi: 10.1371/journal.pone.0328526 (PMC12279097; doi:10.1371/journal.pone.0328526)
Supplement: S6 Table — Temporal joinpoint analysis of ASIR for hematological malignancies in Taiwan (1990 − 2021). (DOCX) [file pone.0328526.s016.docx]

**S6 Table Temporal joinpoint analysis of ASIR for hematological malignancies in Taiwan (1990−2021).**

| Diseases | Start | End | Values | \|Lower | Upper | P | Measures |
| --- | --- | --- | --- | --- | --- | --- | --- |
| ALL | 1990 | 2006 | 1.53 | 1.34 | 1.73 | <0.0001 | APC |
| ALL | 2006 | 2009 | 13.02 | 8.37 | 17.87 | <0.0001 | APC |
| ALL | 2009 | 2018 | 0.00 | −0.49 | 0.50 | 0.9862 | APC |
| ALL | 2018 | 2021 | −3.12 | −5.72 | −0.45 | 0.0246 | APC |
| AML | 1990 | 1994 | 4.78 | 2.72 | 6.88 | 0.0002 | APC |
| AML | 1994 | 2006 | 2.16 | 1.91 | 2.42 | <0.0001 | APC |
| AML | 2006 | 2009 | 3.33 | 0.70 | 6.02 | 0.0162 | APC |
| AML | 2009 | 2015 | −1.32 | −1.89 | −0.74 | 0.0002 | APC |
| AML | 2015 | 2018 | 0.92 | −1.96 | 3.89 | 0.5092 | APC |
| AML | 2018 | 2021 | −2.28 | −3.97 | −0.56 | 0.0129 | APC |
| CLL | 1990 | 1998 | 5.68 | 4.88 | 6.48 | <0.0001 | APC |
| CLL | 1998 | 2010 | 3.83 | 3.51 | 4.15 | <0.0001 | APC |
| CLL | 2010 | 2016 | 1.27 | 0.39 | 2.17 | 0.0070 | APC |
| CLL | 2016 | 2021 | −1.46 | −2.47 | −0.43 | 0.0080 | APC |
| CML | 1990 | 2006 | 0.87 | 0.60 | 1.13 | <0.0001 | APC |
| CML | 2006 | 2010 | 2.58 | 1.05 | 4.13 | 0.0019 | APC |
| CML | 2010 | 2021 | −2.69 | −2.92 | −2.47 | <0.0001 | APC |
| Other leukemia | 1990 | 2006 | 2.15 | 2.00 | 2.30 | <0.0001 | APC |
| Other leukemia | 2006 | 2010 | 5.48 | 3.28 | 7.73 | <0.0001 | APC |
| Other leukemia | 2010 | 2015 | 0.02 | −1.29 | 1.35 | 0.9767 | APC |
| Other leukemia | 2015 | 2021 | −3.8 | −4.52 | −3.07 | <0.0001 | APC |
| HL | 1990 | 1998 | 3.35 | 1.48 | 5.25 | 0.0010 | APC |
| HL | 1998 | 2021 | −1.21 | −1.64 | −0.78 | <0.0001 | APC |
| BL | 1990 | 1997 | 1.94 | 1.50 | 2.38 | <0.0001 | APC |
| BL | 1997 | 2004 | 7.25 | 6.67 | 7.84 | <0.0001 | APC |
| BL | 2004 | 2009 | 3.62 | 2.37 | 4.89 | <0.0001 | APC |
| BL | 2009 | 2021 | −0.4 | −0.67 | −0.14 | 0.0050 | APC |
| Other NHL | 1990 | 1997 | 6.23 | 5.62 | 6.85 | <0.0001 | APC |
| Other NHL | 1997 | 2021 | 0.02 | −0.11 | 0.14 | 0.8050 | APC |
| MM | 1990 | 1992 | 0.08 | −5.81 | 6.33 | 0.9791 | APC |
| MM | 1992 | 1997 | 7.24 | 5.34 | 9.16 | <0.0001 | APC |
| MM | 1997 | 2002 | 2.85 | 1.02 | 4.72 | 0.0046 | APC |
| MM | 2002 | 2005 | −1.61 | −7.23 | 4.35 | 0.5654 | APC |
| MM | 2005 | 2009 | 3.69 | 0.43 | 7.06 | 0.0288 | APC |
| MM | 2009 | 2021 | 0.63 | 0.19 | 1.06 | 0.0074 | APC |
| MD/MP & other HM | 1990 | 2006 | 0.23 | 0.22 | 0.24 | <0.0001 | APC |
| MD/MP & other HM | 2006 | 2010 | 0.56 | 0.43 | 0.69 | <0.0001 | APC |
| MD/MP & other HM | 2010 | 2014 | −0.05 | −0.16 | 0.07 | 0.3911 | APC |
| MD/MP & other HM | 2014 | 2019 | 0.45 | 0.39 | 0.51 | <0.0001 | APC |
| MD/MP & other HM | 2019 | 2021 | −0.04 | −0.23 | 0.15 | 0.6533 | APC |
| ALL | 1990 | 2021 | 1.68 | 1.19 | 2.17 | <0.0001 | AAPC |
| AML | 1990 | 2021 | 1.37 | 0.89 | 1.84 | <0.0001 | AAPC |
| CLL | 1990 | 2021 | 2.93 | 2.61 | 3.25 | <0.0001 | AAPC |
| CML | 1990 | 2021 | −0.20 | −0.43 | 0.04 | 0.1061 | AAPC |
| Other leukemia | 1990 | 2021 | 1.05 | 0.68 | 1.41 | <0.0001 | AAPC |
| HL | 1990 | 2021 | −0.06 | −0.60 | 0.49 | 0.8390 | AAPC |
| BL | 1990 | 2021 | 2.46 | 2.20 | 2.73 | <0.0001 | AAPC |
| Other NHL | 1990 | 2021 | 1.39 | 1.23 | 1.54 | <0.0001 | AAPC |
| MM | 1990 | 2021 | 2.16 | 1.30 | 3.03 | <0.0001 | AAPC |
| MD/MP & other HM | 1990 | 2021 | 0.26 | 0.23 | 0.28 | <0.0001 | AAPC |

ASIR: age-standardized incidence rates; ALL: acute lymphoid leukemia; AML: acute myeloid leukemia, CLL: chronic lymphoid leukemia; CML: chronic myeloid leukemia; HL: Hodgkin lymphoma; BL: Burkitt lymphoma; NHL: non-Hodgkin lymphoma; MM: multiple myeloma; MD/MP & other HN: myelodysplastic, myeloproliferative, and other hematopoietic neoplasms; ASR: age-standardized rates; APC: annual percent change; AAPC: average annual percent change.
