# Supplementary material for: Hematological malignancy burden in mainland China and Taiwan from 1990 to 2021 and decadal projections: Insights from the global burden of disease study 2021
Source: PLoS One. 2025 Jul 21;20(7):e0328526. doi: 10.1371/journal.pone.0328526 (PMC12279097; doi:10.1371/journal.pone.0328526)
Supplement: S7 Table — Temporal joinpoint analysis of ASMR for hematological malignancies in Taiwan (1990 − 2021). (DOCX) [file pone.0328526.s017.docx]

**S7 Table Temporal joinpoint analysis of ASMR for hematological malignancies in Taiwan (1990−2021).**

| Diseases | Start | End | Values | \|Lower | Upper | P | Measures |
| --- | --- | --- | --- | --- | --- | --- | --- |
| ALL | 1990 | 2006 | −0.87 | −1.10 | −0.64 | <0.0001 | APC |
| ALL | 2006 | 2010 | 4.43 | 3.14 | 5.73 | <0.0001 | APC |
| ALL | 2010 | 2015 | −0.33 | −1.05 | 0.40 | 0.3608 | APC |
| ALL | 2015 | 2021 | −3.9 | −4.41 | −3.38 | <0.0001 | APC |
| AML | 1990 | 1994 | 4.17 | 1.12 | 7.32 | 0.0092 | APC |
| AML | 1994 | 2009 | 2.00 | 1.75 | 2.25 | <0.0001 | APC |
| AML | 2009 | 2021 | −1.05 | −1.30 | −0.80 | <0.0001 | APC |
| CLL | 1990 | 2004 | 2.73 | 2.39 | 3.07 | 0.0000 | APC |
| CLL | 2004 | 2015 | 0.78 | 0.39 | 1.17 | 0.0004 | APC |
| CLL | 2015 | 2021 | −3.79 | −4.66 | −2.9 | <0.0001 | APC |
| CML | 1990 | 2011 | −1.08 | −1.25 | −0.91 | <0.0001 | APC |
| CML | 2011 | 2021 | −3.64 | −3.91 | −3.36 | <0.0001 | APC |
| Other leukemia | 1990 | 2007 | 1.89 | 1.72 | 2.06 | <0.0001 | APC |
| Other leukemia | 2007 | 2010 | 5.07 | −0.44 | 10.88 | 0.0699 | APC |
| Other leukemia | 2010 | 2015 | −0.37 | −2.03 | 1.33 | 0.6568 | APC |
| Other leukemia | 2015 | 2021 | −4.71 | −5.56 | −3.84 | <0.0001 | APC |
| HL | 1990 | 2001 | −1.66 | −2.62 | −0.69 | 0.0017 | APC |
| HL | 2001 | 2004 | −10.31 | −22.78 | 4.17 | 0.1466 | APC |
| HL | 2004 | 2021 | −2.74 | −3.31 | −2.16 | <0.0001 | APC |
| BL | 1990 | 1997 | −2.32 | −2.69 | −1.95 | <0.0001 | APC |
| BL | 1997 | 2004 | 3.32 | 2.82 | 3.82 | <0.0001 | APC |
| BL | 2004 | 2010 | 0.06 | −0.70 | 0.82 | 0.8734 | APC |
| BL | 2010 | 2021 | −1.48 | −1.74 | −1.22 | 0.0000 | APC |
| Other NHL | 1990 | 1996 | 4.17 | 3.25 | 5.10 | <0.0001 | APC |
| Other NHL | 1996 | 2021 | −0.72 | −0.85 | −0.6 | <0.0001 | APC |
| MM | 1990 | 1992 | −0.13 | −6.76 | 6.97 | 0.9683 | APC |
| MM | 1992 | 1997 | 6.77 | 4.71 | 8.87 | <0.0001 | APC |
| MM | 1997 | 2002 | 2.66 | 0.76 | 4.60 | 0.0090 | APC |
| MM | 2002 | 2005 | −2.40 | −7.94 | 3.48 | 0.3906 | APC |
| MM | 2005 | 2009 | 2.88 | −0.12 | 5.96 | 0.0586 | APC |
| MM | 2009 | 2021 | 0.19 | −0.22 | 0.61 | 0.3350 | APC |
| MD/MP & other HM | 1990 | 2006 | 1.68 | 1.30 | 2.05 | <0.0001 | APC |
| MD/MP & other HM | 2006 | 2014 | −1.03 | −1.35 | −0.71 | <0.0001 | APC |
| MD/MP & other HM | 2014 | 2017 | 1.72 | −0.60 | 4.09 | 0.1394 | APC |
| MD/MP & other HM | 2017 | 2021 | −1.89 | −3.02 | −0.74 | 0.0026 | APC |
| ALL | 1990 | 2021 | −0.71 | −0.95 | −0.47 | <0.0001 | AAPC |
| AML | 1990 | 2021 | 1.08 | 0.69 | 1.48 | <0.0001 | AAPC |
| CLL | 1990 | 2021 | 0.75 | 0.49 | 1.00 | <0.0001 | AAPC |
| CML | 1990 | 2021 | −1.92 | −2.05 | −1.78 | <0.0001 | AAPC |
| Other leukemia | 1990 | 2021 | 0.51 | −0.07 | 1.10 | 0.0854 | AAPC |
| HL | 1990 | 2021 | −3.12 | −4.51 | −1.71 | <0.0001 | AAPC |
| BL | 1990 | 2021 | −0.31 | −0.52 | −0.1 | 0.0038 | AAPC |
| Other NHL | 1990 | 2021 | 0.21 | 0.02 | 0.40 | 0.0326 | AAPC |
| MM | 1990 | 2021 | 1.69 | 0.82 | 2.57 | 0.0001 | AAPC |
| MD/MP & other HM | 1990 | 2021 | 0.51 | 0.19 | 0.83 | 0.0018 | AAPC |

ASMR: age-standardized mortality rates; ALL: acute lymphoid leukemia; AML: acute myeloid leukemia, CLL: chronic lymphoid leukemia; CML: chronic myeloid leukemia; HL: Hodgkin lymphoma; BL: Burkitt lymphoma; NHL: non-Hodgkin lymphoma; MM: multiple myeloma; MD/MP & other HN: myelodysplastic, myeloproliferative, and other hematopoietic neoplasms; ASR: age-standardized rates; APC: annual percent change; AAPC: average annual percent change.
