# Supplementary material for: Hematological malignancy burden in mainland China and Taiwan from 1990 to 2021 and decadal projections: Insights from the global burden of disease study 2021
Source: PLoS One. 2025 Jul 21;20(7):e0328526. doi: 10.1371/journal.pone.0328526 (PMC12279097; doi:10.1371/journal.pone.0328526)
Supplement: S8 Table — Temporal joinpoint analysis of ASDR for hematological malignancies in Taiwan (1990 − 2021). (DOCX) [file pone.0328526.s018.docx]

**S8 Table Temporal joinpoint analysis of ASDR for hematological malignancies in Taiwan (1990−2021).**

| Diseases | Start | End | Values | \|Lower | Upper | P | Measures |
| --- | --- | --- | --- | --- | --- | --- | --- |
| ALL | 1990 | 2006 | −1.66 | −1.87 | −1.45 | <0.0001 | APC |
| ALL | 2006 | 2009 | 7.05 | 4.58 | 9.59 | <0.0001 | APC |
| ALL | 2009 | 2014 | 0.04 | −0.68 | 0.75 | 0.9151 | APC |
| ALL | 2014 | 2021 | −3.33 | −3.74 | −2.93 | <0.0001 | APC |
| AML | 1990 | 2006 | 0.72 | 0.43 | 1.02 | 0.0001 | APC |
| AML | 2006 | 2009 | 4.22 | 0.21 | 8.41 | 0.0403 | APC |
| AML | 2009 | 2015 | −1.79 | −2.63 | −0.94 | 0.0003 | APC |
| AML | 2015 | 2018 | 1.72 | −2.54 | 6.17 | 0.4128 | APC |
| AML | 2018 | 2021 | −2.35 | −5.08 | 0.45 | 0.0945 | APC |
| CLL | 1990 | 1994 | 3.78 | 1.36 | 6.26 | 0.0036 | APC |
| CLL | 1994 | 2010 | 1.61 | 1.38 | 1.83 | <0.0001 | APC |
| CLL | 2010 | 2017 | 0.55 | −0.11 | 1.22 | 0.1001 | APC |
| CLL | 2017 | 2021 | −2.29 | −3.78 | −0.78 | 0.0050 | APC |
| CML | 1990 | 2006 | −2.23 | −2.51 | −1.95 | <0.0001 | APC |
| CML | 2006 | 2010 | 0.69 | −0.71 | 2.10 | 0.3199 | APC |
| CML | 2010 | 2021 | −3.47 | −3.68 | −3.26 | <0.0001 | APC |
| Other leukemia | 1990 | 1994 | 1.96 | 0.87 | 3.06 | 0.0016 | APC |
| Other leukemia | 1994 | 1997 | −0.5 | −3.56 | 2.65 | 0.7350 | APC |
| Other leukemia | 1997 | 2006 | 0.74 | 0.42 | 1.06 | 0.0002 | APC |
| Other leukemia | 2006 | 2010 | 4.89 | 3.21 | 6.59 | <0.0001 | APC |
| Other leukemia | 2010 | 2015 | −0.15 | −1.16 | 0.87 | 0.7581 | APC |
| Other leukemia | 2015 | 2021 | −3.71 | −4.27 | −3.16 | <0.0001 | APC |
| HL | 1990 | 2001 | −1.65 | −2.66 | −0.64 | 0.0027 | APC |
| HL | 2001 | 2004 | −9.24 | −22.91 | 6.86 | 0.2323 | APC |
| BL | 2004 | 2021 | −2.8 | −3.42 | −2.18 | <0.0001 | APC |
| BL | 1990 | 1997 | −2.61 | −3.07 | −2.15 | <0.0001 | APC |
| BL | 1997 | 2004 | 3.86 | 3.26 | 4.47 | <0.0001 | APC |
| BL | 2004 | 2011 | 0.20 | −0.48 | 0.88 | 0.5479 | APC |
| BL | 2011 | 2021 | −2.13 | −2.48 | −1.79 | <0.0001 | APC |
| Other NHL | 1990 | 1996 | 3.49 | 2.62 | 4.37 | <0.0001 | APC |
| Other NHL | 1996 | 2008 | −1.67 | −2.01 | −1.32 | <0.0001 | APC |
| Other NHL | 2008 | 2021 | −0.43 | −0.79 | −0.06 | 0.0233 | APC |
| MM | 1990 | 1992 | −0.92 | −7.61 | 6.26 | 0.7819 | APC |
| MM | 1992 | 1997 | 6.82 | 4.67 | 9.02 | <0.0001 | APC |
| MM | 1997 | 2002 | 1.71 | −0.23 | 3.69 | 0.0797 | APC |
| MM | 2002 | 2005 | −2.31 | −7.98 | 3.71 | 0.4186 | APC |
| MM | 2005 | 2009 | 2.43 | −0.49 | 5.44 | 0.0973 | APC |
| MM | 2009 | 2021 | 0.57 | 0.16 | 0.97 | 0.0094 | APC |
| MD/MP & other HM | 1990 | 2007 | 1.24 | 0.98 | 1.50 | <0.0001 | APC |
| MD/MP & other HM | 2007 | 2014 | −1.36 | −1.72 | −1.00 | <0.0001 | APC |
| MD/MP & other HM | 2014 | 2017 | 0.84 | −1.37 | 3.09 | 0.4420 | APC |
| MD/MP & other HM | 2017 | 2021 | −1.28 | −2.27 | −0.28 | 0.0144 | APC |
| ALL | 1990 | 2021 | −0.96 | −1.23 | −0.69 | <0.0001 | AAPC |
| AML | 1990 | 2021 | 0.36 | −0.26 | 0.98 | 0.2587 | AAPC |
| CLL | 1990 | 2021 | 1.13 | 0.74 | 1.52 | <0.0001 | AAPC |
| CML | 1990 | 2021 | −2.30 | −2.53 | −2.07 | <0.0001 | AAPC |
| Other leukemia | 1990 | 2021 | 0.28 | −0.14 | 0.69 | 0.1921 | AAPC |
| HL | 1990 | 2021 | −3.04 | −4.56 | −1.50 | 0.0001 | AAPC |
| BL | 1990 | 2021 | −0.39 | −0.63 | −0.15 | 0.0014 | AAPC |
| Other NHL | 1990 | 2021 | −0.17 | −0.41 | 0.08 | 0.1906 | AAPC |
| MM | 1990 | 2021 | 1.59 | 0.71 | 2.49 | 0.0004 | AAPC |
| MD/MP & other HM | 1990 | 2021 | 0.28 | <0.01 | 0.56 | 0.0507 | AAPC |

ASDR: age-standardized DALYs rates; ALL: acute lymphoid leukemia; AML: acute myeloid leukemia, CLL: chronic lymphoid leukemia; CML: chronic myeloid leukemia; HL: Hodgkin lymphoma; BL: Burkitt lymphoma; NHL: non-Hodgkin lymphoma; MM: multiple myeloma; MD/MP & other HN: myelodysplastic, myeloproliferative, and other hematopoietic neoplasms; ASR: age-standardized rates; APC: annual percent change; AAPC: average annual percent change.
