# Supplementary material for: Hematological malignancy burden in mainland China and Taiwan from 1990 to 2021 and decadal projections: Insights from the global burden of disease study 2021
Source: PLoS One. 2025 Jul 21;20(7):e0328526. doi: 10.1371/journal.pone.0328526 (PMC12279097; doi:10.1371/journal.pone.0328526)
Supplement: S9 Table — (DOCX) [file pone.0328526.s019.docx]

**S9 Table Predicted incidence and mortality of hematological malignancies in mainland China.**

|  | **Incidence rates per 100,000 person (95% UI)** | | **Mortality rates per 100,000 person (95% UI)** | | |
| --- | --- | --- | --- | --- | --- |
|  | **2022** | **2035** | | **2022** | **2035** |
| ALL |  |  | |  |  |
| Male | 11.95 (10.50−13.41) | 11.64 (4.76−18.52) | | 2.09 (1.95−2.24) | 1.28 (0.80−1.76) |
| Female | 8.89 (7.89−9.89) | 6.38 (2.75−10.01) | | 1.41 (1.31−1.52) | 0.83 (0.57−1.10) |
| AML |  |  | |  |  |
| Male | 1.34 (1.25−1.42) | 1.12 (0.68−1.56) | | 1.15 (1.07−1.22) | 0.90 (0.52−1.27) |
| Female | 1.13 (1.06−1.19) | 1.05 (0.59−1.51) | | 0.90 (0.85−0.95) | 0.79 (0.42−1.15) |
| CLL |  |  | |  |  |
| Male | 1.65 (1.57−1.73) | 1.80 (1.17−2.42) | | 0.83 (0.80−0.87) | 0.74 (0.50−0.99) |
| Female | 1.15 (1.08−1.21) | 1.24 (0.86−1.61) | | 0.45 (0.43−0.47) | 0.40 (0.24−0.56) |
| CML |  |  | |  |  |
| Male | 0.25 (0.23−0.26) | 0.25 (0.15−0.34) | | 0.11 (0.10−0.12) | 0.08 (0.04−0.13) |
| Female | 0.18 (0.17−0.19) | 0.17 (0.09−0.24) | | 0.08 (0.07−0.09) | 0.06 (0.02−0.10) |
| Other leukemia |  |  | |  |  |
| Male | 1.35 (1.28−1.42) | 1.07 (0.76−1.39) | | 0.77 (0.74−0.80) | 0.59 (0.41−0.77) |
| Female | 0.79 (0.75−0.82) | 0.64 (0.46−0.82) | | 0.50 (0.48−0.51) | 0.40 (0.29−0.52) |
| HL |  |  | |  |  |
| Male | 0.30 (0.28−0.31) | 0.29 (0.17−0.40) | | 0.15 (0.14−0.16) | 0.12 (0.06−0.18) |
| Female | 0.19 (0.17−0.20) | 0.20 (0.12−0.28) | | 0.09 (0.09−0.10) | 0.08 (0.04−0.12) |
| BL |  |  | |  |  |
| Male | 0.14 (0.12−0.15) | 0.20 (0.08−0.31) | | 0.02 (0.02−0.03) | 0.02 (0.01−0.04) |
| Female | 0.07 (0.06−0.08) | 0.11 (0.02−0.20) | | 0.01 (0.01−0.01) | 0.01 (<0.01−0.03) |
| Other NHL |  |  | |  |  |
| Male | 7.23 (6.61−7.85) | 6.07 (1.67−10.46) | | 2.65 (2.45−2.85) | 1.75 (0.54−2.95) |
| Female | 3.98 (3.75−4.22) | 4.62 (1.20−8.04) | | 1.46 (1.39−1.53) | 1.42 (0.49−2.34) |
| MM |  |  | |  |  |
| Male | 1.03 (0.96−1.10) | 1.22 (<0.01−2.56) | | 1.13 (1.05−1.21) | 1.20 (<0.01−2.55) |
| Female | 0.59 (0.55−0.64) | 0.73 (<0.01−1.71) | | 0.71 (0.65−0.77) | 0.83 (<0.01−1.98) |
| MD/MP & other HM |  |  | |  |  |
| Male | 5.75 (5.33−6.17) | 5.54 (3.96−7.13) | | 0.31 (0.30−0.33) | 0.28 (0.18−0.38) |
| Female | 3.32 (3.07−3.56) | 3.29 (2.24−4.34) | | 0.19 (0.18−0.21) | 0.21 (0.13−0.30) |

ALL: acute lymphoid leukemia; AML: acute myeloid leukemia, CLL: chronic lymphoid leukemia; CML: chronic myeloid leukemia; HL: Hodgkin lymphoma; BL: Burkitt lymphoma; NHL: non-Hodgkin lymphoma; MM: multiple myeloma; MD/MP & other HN: myelodysplastic, myeloproliferative, and other hematopoietic neoplasms; UI: uncertainty interval.
