# Supplementary material for: Hematological malignancy burden in mainland China and Taiwan from 1990 to 2021 and decadal projections: Insights from the global burden of disease study 2021
Source: PLoS One. 2025 Jul 21;20(7):e0328526. doi: 10.1371/journal.pone.0328526 (PMC12279097; doi:10.1371/journal.pone.0328526)
Supplement: S10 Table — (DOCX) [file pone.0328526.s020.docx]

**S10 Table Predicted incidence and mortality rates of hematological malignancies in Taiwan province.**

|  | **Incidence rates per 100,000 person (95% UI)** | | **Mortality rates per 100,000 person (95% UI)** | | |
| --- | --- | --- | --- | --- | --- |
|  | **2022** | **2035** | | **2022** | **2035** |
| ALL |  |  | |  |  |
| Male | 2.74 (2.19−3.29) | 2.49 (0.31−4.67) | | 0.55 (0.45−0.65) | 0.41 (0.11−0.70) |
| Female | 2.82 (2.22−3.42) | 2.93 (0.52−5.34) | | 045 (0.36−0.64) | 0.39 (0.13−0.65) |
| AML |  |  | |  |  |
| Male | 3.15 (2.88−3.41) | 2.83 (1.57−4.09) | | 2.73 (2.49−2.96) | 2.38 (1.32−3.45) |
| Female | 2.20 (1.99−2.42) | 1.96 (1.08−2.85) | | 1.75 (1.57−1.93) | 1.50 (0.82−2.18) |
| CLL |  |  | |  |  |
| Male | 0.81 (0.70−0.91) | 0.81 (0.30−1.32) | | 0.35 (0.28−0.42) | 0.30 (0.09−0.51) |
| Female | 0.44 (0.37−0.50) | 0.49 (0.17−0.81) | | 0.15 (0.11−0.19) | 0.16 (0.02−0.29) |
| CML |  |  | |  |  |
| Male | 0.59 (0.50−0.67) | 0.45 (0.17−0.73) | | 0.28 (0.24−0.33) | 0.21 (0.09−0.33) |
| Female | 0.27 (0.22−0.32) | 0.19 (0.06−0.32) | | 0.11 (0.08−0.14) | 0.07 (0.02−0.12) |
| Other leukemia |  |  | |  |  |
| Male | 0.51 (0.41−0.61) | 0.41 (0.09−0.73) | | 0.34 (0.28−0.40) | 0.25 (0.06−0.45) |
| Female | 0.20 (0.16−0.25) | 0.16 (0.03−0.30) | | 0.17 (0.13−0.20) | 0.13 (0.04−0.22) |
| HL |  |  | |  |  |
| Male | 0.53 (0.45−0.61) | 0.41 (0.16−0.66) | | 0.05 (0.03−0.06) | 0.03 (0.01−0.05) |
| Female | 0.24 (0.19−0.29) | 0.17 (0.04−0.29) | | 0.02 (0.01−0.03) | 0.01 (<0.01−0.02) |
| BL |  |  | |  |  |
| Male | 0.19 (0.13−0.25) | 0.17 (<0.01−0.38) | | <0.01 (<0.01−<0.01) | <0.01 (<0.01−<0.01) |
| Female | 0.05 (0.01−0.08) | 0.03 (<0.01−0.16) | | <0.01 (<0.01−<0.01) | <0.01 (<0.01−<0.01) |
| Other NHL |  |  | |  |  |
| Male | 8.86 (8.33−9.40) | 7.86 (4.17−11.55) | | 4.48 (4.17−4.78) | 3.86 (2.17−5.56) |
| Female | 5.02 (4.63−5.41) | 4.55 (1.85−7.26) | | 2.47 (2.26−2.68) | 2.08 (0.97−3.19) |
| MM |  |  | |  |  |
| Male | 2.40 (2.20−2.60) | 2.42 (1.36−3.48) | | 2.41 (2.18−2.63) | 2.34 (1.27−3.41) |
| Female | 1.60 (1.45−1.76) | 1.64 (0.74−2.55) | | 1.52 (1.35−1.68) | 1.58 (0.71−2.44) |
| MD/MP & other HM |  |  | |  |  |
| Male | 3.46 (3.18−3.74) | 3.78 (2.39−5.18) | | 0.56 (0.49−0.63) | 0.55 (0.27−0.82) |
| Female | 2.41 (2.17−2.64) | 2.66 (1.60−3.73) | | 0.21 (0.18−0.25) | 0.19 (0.08−0.31) |

ALL: acute lymphoid leukemia; AML: acute myeloid leukemia, CLL: chronic lymphoid leukemia; CML: chronic myeloid leukemia; HL: Hodgkin lymphoma; BL: Burkitt lymphoma; NHL: non-Hodgkin lymphoma; MM: multiple myeloma; MD/MP & other HN: myelodysplastic, myeloproliferative, and other hematopoietic neoplasms; UI: uncertainty interval.
